# Supplementary material for: Characterisation of pathogen-specific regions and novel effector candidates in Fusarium oxysporum f. sp. cepae
Source: Sci Rep. 2018 Sep 10;8:13530. doi: 10.1038/s41598-018-30335-7 (PMC6131394; doi:10.1038/s41598-018-30335-7)
Supplement: Supplementary file 1 — Supplementary Information [file 41598_2018_30335_MOESM1_ESM.pdf]

Supplementary Information

Characterisation of pathogen-specific regions and novel effector candidates in *Fusarium oxysporum* f. sp. *Cepae*

Andrew D. Armitage[1], Andrew Taylor[2], Maria K. Sobczyk[1], Laura Baxter[2], Bethany P.J. Greenfield[1], Helen J. Bates[1], Fiona Wilson[1], Alison C. Jackson[2], Sascha Ott[3], Richard J. Harrison[1]* and John P. Clarkson[2]

1. NIAB-EMR, New Road, East Malling, Kent, ME19 6BJ, UK

2. Warwick Crop Centre, School of Life Sciences, University of Warwick, Wellesbourne, Warwick, CV35 9EF, UK

3. Department of Computer Science, University of Warwick, CV4 7AL, UK

*Correspondence: Email: richard.harrison@emr.ac.uk

Supplementary Legends

Supplementary Table 1
Accession numbers of *Fusarium oxysporum* (Fo) and *F. oxysporum* f. sp *cepae* (Foc) assemblies and gene models deposited at Genbank as Whole Genome Shotgun projects.

Supplementary Table 2
Identification of core (C), lineage specific (LS), and pathogen specific (PS) regions in *Fusarium oxysporum* f. sp. *lycopersici* (Fol) genome, through alignment of *F. oxysporum* (Fo) and *F. oxysporum* f. sp. *cepae* (Foc) assemblies. The percentage of non-masked bp covered by aligned sequence is shown for each reference contig.

Supplementary Table 3
Distribution of features throughout the *Fusarium oxysporum* f. sp. *cepae* Fus2 genome. Numbers and density of genes predicted as secreted proteins, secreted carbohydrate active enzymes (CAZY), and secreted proteins with an effector-like structure (as predicted by EffectorP). Number and density of secondary metabolite clusters (Sec. Met. clusters) and MIMPs are also shown.

Supplementary Table 4
Gene ID and ortholog groups of previously identified *Fusarium oxysporum* f. sp. *lycopersici* (Fol) genes Secreted In Xylem (*SIX*) pathogenicity genes following BLAST searches against *F. oxysporum* f. sp. *cepae* (Foc) Fus2 genome. Genes with no identifiable orthologs are marked as singletons. * Evidence for an additional unpredicted *SIX3* gene was found in the Foc A23 assembly, spanning a contig break.

Supplementary Table 5
ID, location, orthogroup, effector evidence, expression in planta and functional annotation for all genes predicted in pathogenic *Fusarium oxysporum* f. sp. *cepae* (Foc) isolate Fus2. Orthogroup contents show the number of proteins clustered into a particular orthogroup by isolate including pathogenic Foc isolates (Fus2, A23, 125), non-pathogenic *F. oxysporum* (Fo) isolates from onion (A13, PG, A28, CB3) and reference genomes for non-pathogenic Fo (isolate Fo47) and F. oxysporum f. sp. *lycopersici* (isolate 4287).
.

Supplementary Table 6
SNP variants observed in secreted effector candidates located in pathogen-specific regions of *Fusarium oxysporum* f. sp. *cepae* (Foc) isolate Fus2, through comparison to other Foc isolates.

Supplementary Table 7
Comparison of distribution of codon bias across different subsets of genes relative to all genes (t-test), calculated over: all, core, all lineage-specific (LS) chromosomes, LS chromosomes identified as pathogen-specific (PS) and LS chromosomes not identified as PS (non-PS).

Supplementary Table 8
Enriched Interproscan terms associated with predicted proteins encoded on(A) pathogen specific (PS) and B) non-PS lineage specific (LS) regions in comparison to core regions. Annotation shown are those significant following Benjamini-Hochberg correction. Terms shown in bold are of particular interest as they were not significantly enriched in other LS regions, or nearly significant (P-values < 0.05 but failed multiple test correction) in other LS regions.

Supplementary Table 9
Expression values (fpkm) and annotations of the 50 highest expressed core genes of *F. oxysporum* f. sp. *cepae* Fus2 and those lineage specific (LS) and pathogen-specific (PS) genes showing equal or greater expression.

Supplementary Table 10
Identification of transcription factors within the genome of *Fusarium oxysporum* f. sp. *cepae* isolate Fus2 and their expression *in planta*. A) Transcription factors identified in pathogen specific (PS) and non-PS lineage specific (LS) regions. B) Homologs to known *F. oxysporum* f. sp. *lycopersici* transcription factors TF1-9 (B).

Supplementary Figure 1
Duplication of genes between and within Fus2 chromosomes show different patterns between core, effector-enriched and LS regions. Links are shown between genes on a Foc contig and all other genes in that orthology group. Genes on core chromosomes (1, 2, 4, 5, 7, 8, 9, 10) do not show high levels of gene duplication across their entire length, with genes in shared ortholog groups primarily on telomeric regions and shared with telomeric regions of other chromosomes. Examples are provided for chromosomes 1 (a) and 4 (b). Duplicated genes on LS regions show a bias towards being shared with other LS regions. Effector enriched core chromosomes (11, 12, 13) show a greater numbers of duplications and are more likely to share genes with one another, as observed in chromosome 11 (c). Genes on Foc LS regions share large numbers of duplicated across their entire length, with duplications primarily shared with other LS regions. Example provided for LS contig 10 (d).

Supplementary Figure 2
Repeat masked and gene-coding content of chromosomes and LS regions within Fol and Foc chromosomes / LS regions. Percentage of bp repeat masked in Fol (A) and Foc core (green), non-PS LS (orange) and PS (red) regions (B). Gene density is shown for Fol (C) and Foc (D) core, LS and PS regions. Shaded bars indicate the contribution of genes located in repeatmasked regions towards gene density

Supplementary Table 1

Accession numbers of *Fusarium oxysporum* (Fo) and *F. oxysporum* f. sp *cepae* (Foc) assemblies and gene models deposited at Genbank as Whole Genome Shotgun projects.

Organism	Isolate	Accession number	Version number
Foc	Fus2	MRCU00000000	MRCU01000000
Foc	125	MRCV00000000	MRCV01000000
Foc	A23	MRCW00000000	MRCW01000000
Fo	A13	MRCX00000000	MRCX01000000
Fo	A28	MRCY00000000	MRCY01000000
Fo	CB3	MRCZ00000000	MRCZ01000000
Fo	PG	MRDA00000000	MRDA01000000

Supplementary Table 2

Identification of core (C), lineage specific (LS), and pathogen specific (PS) regions in *Fusarium oxysporum* f. sp. *lycopersici* (Fol) genome, through alignment of *F. oxysporum* (Fo) and *F. oxysporum* f. sp. *cepae* (Foc) assemblies. The percentage of non-masked bp covered by aligned sequence is shown for each reference contig.

Alignment to Fol chromosome	Specificity	Fol 4287	Foc Fus2	Foc 125	Foc A23	Fo A13	Fo A28	Fo CB3	Fo PG	Fo fo47
1	C	96	90	90	90	90	91	89	91	91
2	C	100	90	91	91	93	92	89	92	92
3	LS	56	6	9	9	13	13	12	11	7
4	C	100	96	96	96	93	95	95	95	96
5	C	100	95	96	96	96	92	96	95	96
6	LS	59	8	10	10	9	7	6	5	5
7	C	100	96	96	96	96	97	96	83	96
8	C	100	96	96	96	96	95	96	97	95
9	C	100	94	97	97	94	94	93	95	92
10	C	100	94	94	94	93	93	93	95	95
11	C	100	96	96	95	94	94	95	97	96
12	C	100	85	89	89	91	90	90	91	89
13	C	100	95	95	95	94	94	94	96	95
14	LS + PS	97	15	29	30	22	18	17	14	8
15	LS	57	7	9	9	12	13	6	10	14

1-25 %
26-50 %
51-75 %
76-100 %

Supplementary Table 3

Distribution of features throughout the *Fusarium oxysporum* f. sp. *cepae* Fus2 genome. Numbers and density of genes predicted as secreted proteins, secreted carbohydrate active enzymes (CAZY), and secreted proteins with an effector-like structure (as predicted by EffectorP). Number and density of secondary metabolite clusters (Sec. Met. clusters) and MIMPs are also shown.

Chromosome / contig	Region	Chromosome length	Total genes	Secreted	CAZY	Eff. like structure	Sec. Met. clusters	MIMPs	Secreted (genes/Mb)	CAZY (genes/Mb)	Eff. like structure (genes/Mb)	Sec. Met. (clusters/Mb)	MIMPs (MIMPs/Mb)
1	Core	6433760	2217	109	25	30	4	0	16.94	3.89	4.66	0.62	0.00
2	Core	4147034	1718	128	30	31	4	2	30.87	7.23	7.48	0.96	0.48
4	Core	5388197	2027	170	38	27	3	0	31.55	7.05	5.01	0.56	0.00
5	Core	5171523	1764	117	27	27	6	0	22.62	5.22	5.22	1.16	0.00
7	Core	4882972	1716	103	26	31	5	0	21.09	5.32	6.35	1.02	0.00
8	Core	4140032	1421	98	25	26	4	0	23.67	6.04	6.28	0.97	0.00
9	Core	3815037	1299	82	21	19	2	1	21.49	5.50	4.98	0.52	0.26
10	Core	3356772	1185	112	28	23	2	1	33.37	8.34	6.85	0.60	0.30
11	Eff. rich	3130441	1262	151	51	36	4	1	48.24	16.29	11.50	1.28	0.32
12	Eff. rich	2929571	1148	186	65	37	6	5	63.49	22.19	12.63	2.05	1.71
13	Eff. rich	3067455	1100	118	32	28	6	3	38.47	10.43	9.13	1.96	0.98
Contig 14	non-PS LS	1115003	390	12	3	4	0	7	10.76	2.69	3.59	0.00	6.28
Contig 20	non-PS LS	452227	155	6	2	2	0	13	13.27	4.42	4.42	0.00	28.75
Contig 22	non-PS LS	181933	65	6	2	0	0	0	32.98	10.99	0.00	0.00	0.00
Contig 16	PS	2313841	713	41	6	14	0	28	17.72	2.59	6.05	0.00	12.10
Contig 10	PS	818454	292	9	2	6	2	28	11.00	2.44	7.33	2.44	34.21
Contig 19	PS	480286	181	17	2	9	1	30	35.40	4.16	18.74	2.08	62.46
Contig 21	PS	331997	130	7	1	5	1	34	21.08	3.01	15.06	3.01	102.41

Supplementary Table 4

Gene ID and ortholog groups of previously identified *Fusarium oxysporum* f. sp. *lycopersici* (Fol) genes Secreted In Xylem (*SIX*) pathogenicity genes following BLAST searches against *F. oxysporum* f. sp. *cepae* (Foc) Fus2 genome. Genes with no identifiable orthologs are marked as singletons. * Evidence for an additional unpredicted *SIX3* gene was found in the Foc A23 assembly, spanning a contig break.

SIX gene	FoL gene	Fus2 gene	Fus2 contig	Orthogroup	
SIX1	FOXG_16418	-	-	singleton	-
SIX2	FOXG_16416	-	-	singleton	-
SIX3	FOXG_16398	BFJ65_g18321	Contig 19 (PS)	orthogroup12653	125(2):A23(1*):Fus2(2):CB3(0):A13(0):A28(0):PG(0):fo47(0):4287(1)
		BFJ65_ g17808	Contig 16 (PS)	orthogroup12653	125(2):A23(1*):Fus2(2):CB3(0):A13(0):A28(0):PG(0):fo47(0):4287(1)
SIX4	-	-	-	-	-
SIX5	not annotated	BFJ65_g18413	Contig 19 (PS)	orthogroup13175	125(1):A23(1):Fus2(1):CB3(0):A13(0):A28(0):PG(0):fo47(0):4287(2)
SIX6	FOXG_14246	-	-	-	-
SIX7	not annotated	BFJ65_g18647	Contig 21 (PS)	orthogroup13885	125(1):A23(1):Fus2(1):CB3(0):A13(0):A28(0):PG(0):fo47(0):4287(0)
SIX8	not annotated	-	-	-	-
SIX9	FOXG_14223	BFJ65_g17715	Contig 16 (PS)	orthogroup12321	125(2):A23(2):Fus2(2):CB3(0):A13(0):A28(0):PG(1):fo47(0):4287(0)
		BFJ65_g14764	Contig 10 (PS)	orthogroup12321	125(2):A23(2):Fus2(2):CB3(0):A13(0):A28(0):PG(1):fo47(0):4287(0)
SIX10	FOXG_17457	BFJ65_g18692	Contig 21 (PS)	orthogroup13429	125(1):A23(1):Fus2(1):CB3(0):A13(0):A28(0):PG(0):fo47(0):4287(1)
SIX11	not annotated	-	-	-	-
SIX12	not annotated	BFJ65_g18627	Contig 21 (PS)	orthogroup14046	125(1):A23(1):Fus2(1):CB3(0):A13(0):A28(0):PG(0):fo47(0):4287(0)
SIX13	not annotated	-	-	-	-
SIX14	not annotated	BFJ65_g18325	Contig 19 (PS)	orthogroup13909	125(1):A23(1):Fus2(1):CB3(0):A13(0):A28(0):PG(0):fo47(0):4287(0)

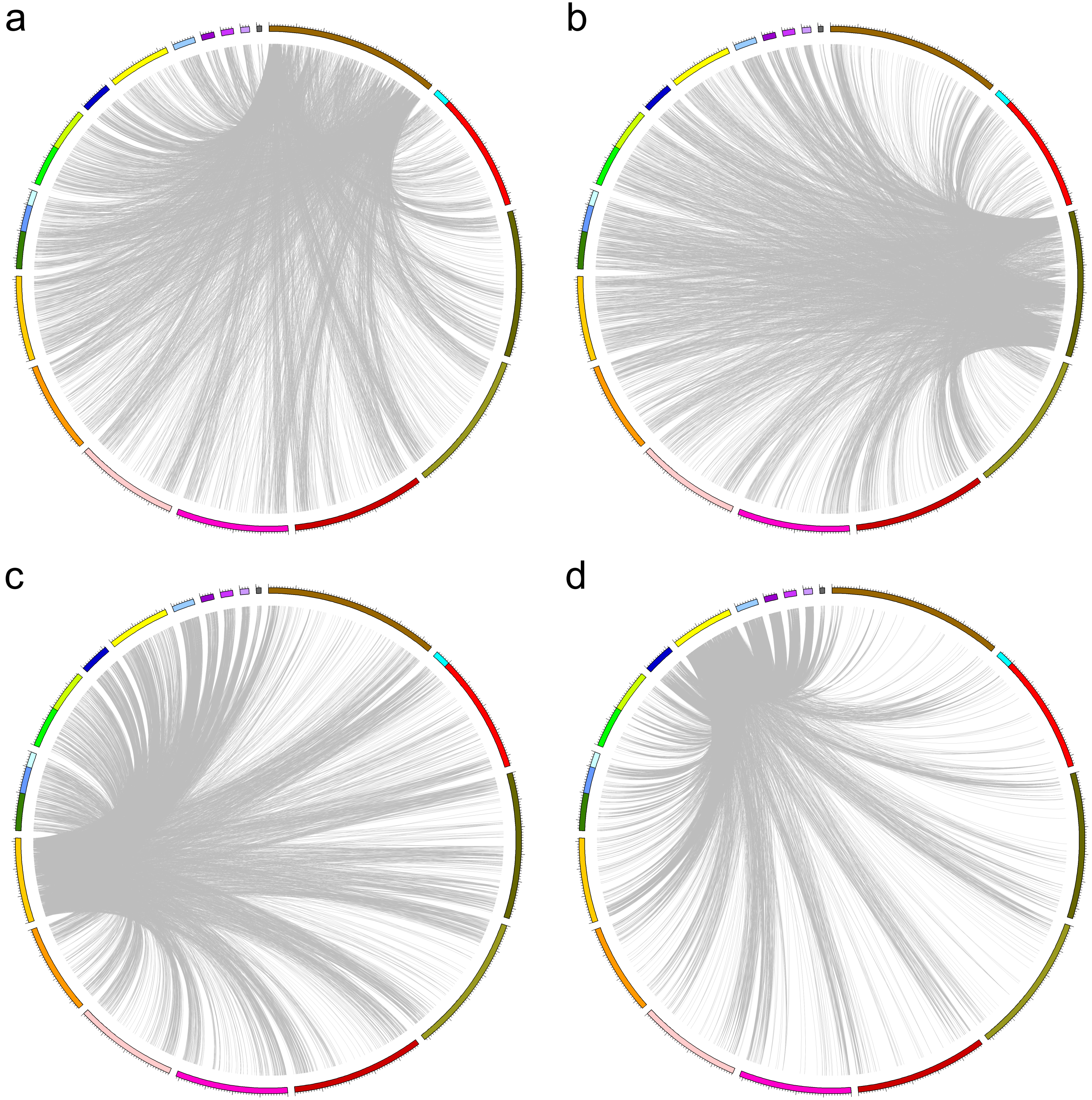

Supplementary Figure 1

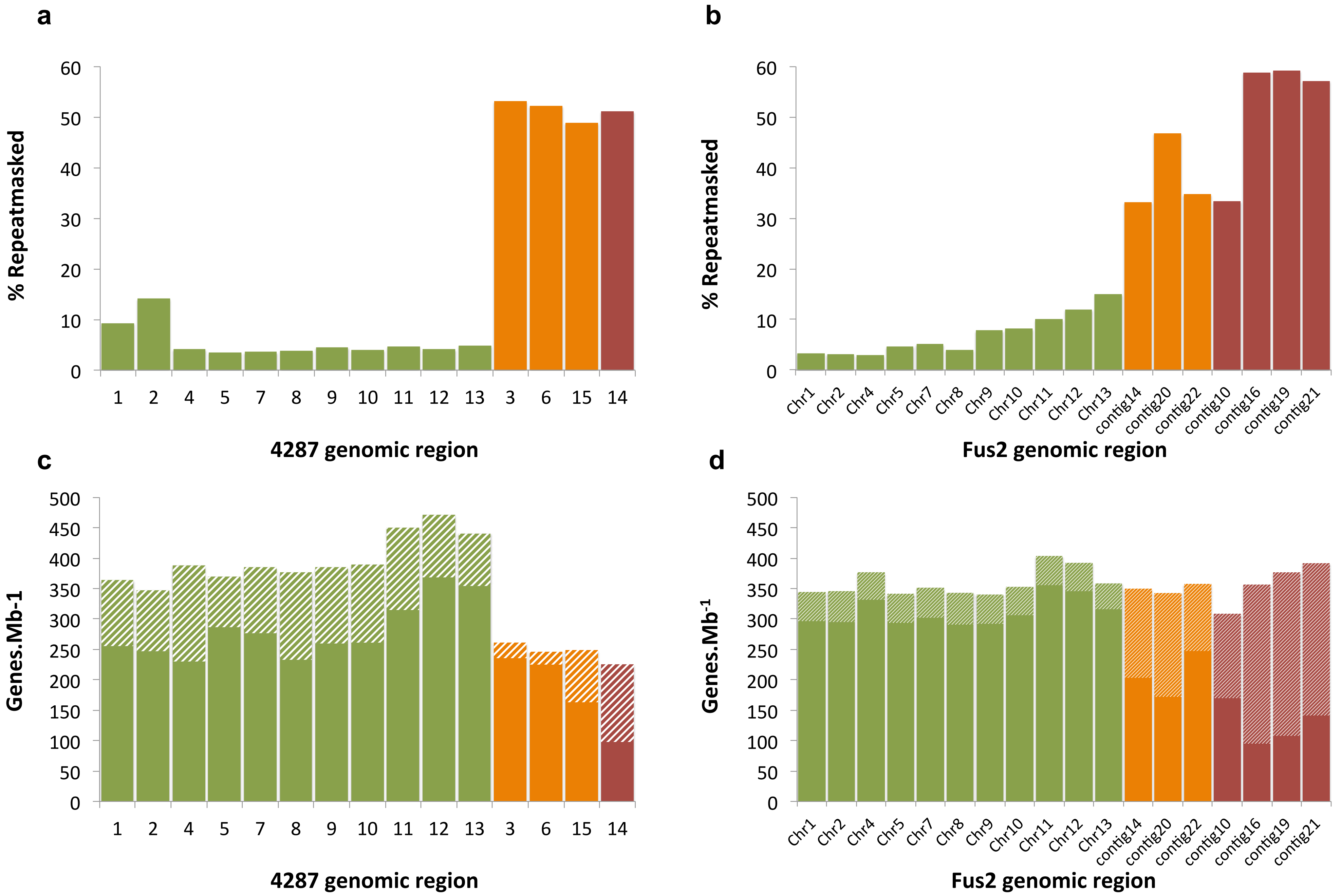

Supplementary Figure 2
